# COVID-19 Diagnosis and Risk of Death Among Adults With Cancer in Indiana: Retrospective Cohort Study

**DOI:** 10.2196/35310

**Published:** 2022-10-06

**Authors:** Nimish Valvi, Hetvee Patel, Giorgos Bakoyannis, David A Haggstrom, Sanjay Mohanty, Brian E Dixon

**Affiliations:** 1 Center for Biomedical Informatics Regenstrief Institute Indianapolis, IN United States; 2 Richard M. Fairbanks School of Public Health Indiana University Indianapolis, IN United States; 3 Center for Health Information and Communication, Health Services Research & Development Service Richard L. Roudebush VA Medical Center Veterans Health Administration Indianapolis, IN United States; 4 Department of Surgery Indiana University School of Medicine Indianapolis, IN United States; 5 Center for Health Services Research Regenstrief Institute Indianapolis, IN United States

**Keywords:** COVID-19, SARS-CoV-2, coronavirus, cancer, survival, mortality, death, oncology, cancer experience, outcome, electronic health record, EHR, patient with cancer, cancer population, Kaplan-Meier, Cox proportional hazards model, hazard ratio, risk

## Abstract

**Background:**

Prior studies, generally conducted at single centers with small sample sizes, found that individuals with cancer experience more severe outcomes due to COVID-19, caused by SARS-CoV-2 infection. Although early examinations revealed greater risk of severe outcomes for patients with cancer, the magnitude of the increased risk remains unclear. Furthermore, prior studies were not typically performed using population-level data, especially those in the United States. Given robust prevention measures (eg, vaccines) are available for populations, examining the increased risk of patients with cancer due to SARS-CoV-2 infection using robust population-level analyses of electronic medical records is warranted.

**Objective:**

The aim of this paper is to evaluate the association between SARS-CoV-2 infection and all-cause mortality among recently diagnosed adults with cancer.

**Methods:**

We conducted a retrospective cohort study of newly diagnosed adults with cancer between January 1, 2019, and December 31, 2020, using electronic health records linked to a statewide SARS-CoV-2 testing database. The primary outcome was all-cause mortality. We used the Kaplan-Meier estimator to estimate survival during the COVID-19 period (January 15, 2020, to December 31, 2020). We further modeled SARS-CoV-2 infection as a time-dependent exposure (immortal time bias) in a multivariable Cox proportional hazards model adjusting for clinical and demographic variables to estimate the hazard ratios (HRs) among newly diagnosed adults with cancer. Sensitivity analyses were conducted using the above methods among individuals with cancer-staging information.

**Results:**

During the study period, 41,924 adults were identified with newly diagnosed cancer, of which 2894 (6.9%) tested positive for SARS-CoV-2. The population consisted of White (n=32,867, 78.4%), Black (n=2671, 6.4%), Hispanic (n=832, 2.0%), and other (n=5554, 13.2%) racial backgrounds, with both male (n=21,354, 50.9%) and female (n=20,570, 49.1%) individuals. In the COVID-19 period analysis, after adjusting for age, sex, race or ethnicity, comorbidities, cancer type, and region, the risk of death increased by 91% (adjusted HR 1.91; 95% CI 1.76-2.09) compared to the pre–COVID-19 period (January 1, 2019, to January 14, 2020) after adjusting for other covariates. In the adjusted time-dependent analysis, SARS-CoV-2 infection was associated with an increase in all-cause mortality (adjusted HR 6.91; 95% CI 6.06-7.89). Mortality increased 2.5 times among adults aged 65 years and older (adjusted HR 2.74; 95% CI 2.26-3.31) compared to adults 18-44 years old, among male (adjusted HR 1.23; 95% CI 1.14-1.32) compared to female individuals, and those with ≥2 chronic conditions (adjusted HR 2.12; 95% CI 1.94-2.31) compared to those with no comorbidities. Risk of mortality was 9% higher in the rural population (adjusted HR 1.09; 95% CI 1.01-1.18) compared to adult urban residents.

**Conclusions:**

The findings highlight increased risk of death is associated with SARS-CoV-2 infection among patients with a recent diagnosis of cancer. Elevated risk underscores the importance of adhering to social distancing, mask adherence, vaccination, and regular testing among the adult cancer population.

## Introduction

As of July 2022, there have been over 1 million deaths from COVID-19 in the United States [1]. Certain patient subgroups, such as older adults, as well as individuals with chronic conditions such as hypertension, diabetes, and chronic lung diseases, have been shown to be at increased risk of morbidity and mortality due to COVID-19 [2]. Given immunosuppression due to both disease and treatment among patients with cancer, there exists increasing risks among patients with cancer who have COVID-19, require hospitalization and intensive care, and are at risk of death [3-5].

As a precaution because of increasing transmission, cancer care providers made sweeping changes to the management of patients with cancer at the start of the pandemic, with changes to radiation therapy sessions, immunotherapy, and administration of oral medications instead of intravenous chemotherapy [6-9]. These changes were introduced based on early evidence, primarily drawn from outside the United States. Early studies from China [10-12] reported a 2- to 4-fold increase in mortality due to COVID-19 among patients with cancer compared to those without cancer, while a few smaller studies reported a case-fatality rate of 29% [13] and poorer outcomes [3] among patients with cancer who have COVID-19. Some studies from Europe and the United States have investigated mortality among cancer subpopulations and demonstrated a 34% [14] increase among individuals with solid tumors compared to hematologic tumors. A retrospective cohort study from the United States found a 2.5-fold increase in hospitalizations among patients with hematologic tumors compared to solid tumors and a 67% increase in hospitalization among adults 65 years and older compared to those 65 years and younger [15].

Prior studies in the United States were generally conducted at single institutions, most with small sample sizes that focused on case-fatality rates [4,16]. Some of the larger studies conducted in the United Kingdom evaluated case fatality rates [17] and cross-sectionally compared all-cause mortality among individuals with active cancer treatment [18,19]. However, these studies did not report negative outcomes because of anticancer therapies, suggesting mortality is driven by demographic factors and comorbidities [18,19]. Furthermore, prior studies [4,12,15,20] generally examined in populations with COVID-19 severe disease following infection with the SARS-CoV-2 virus. These studies were important to our understanding of the novel COVID-19 as they were conducted within the first 6 months of the pandemic. Now that more than 2 years have passed since the start of the pandemic, it is important to conduct larger retrospective analyses to better understand risks of COVID-19 on mortality among individuals with cancer. As additional protections against COVID-19 become widespread, such as effective vaccines for individuals with immunosuppressive conditions [21-26], patients with cancer should be aware of their risks to make informed choices about prevention and treatment.

The objective of this study is to estimate risk of SARS-CoV-2 infection and death in a large, statewide cohort of adults recently diagnosed with cancer. This study is unique in its use of a population approach to estimating risk, as opposed to earlier studies from a single institution or network of hospitals in a city or region. The study is further unique in its use of infection-based risk rather than examining patients who present for care to a clinic or hospital. Comprehensive cancer diagnosis captured from electronic medical records linked to governmental, hospital-based, and private SARS-CoV-2 testing centers enabled robust data capture for examining population-level risk.

## Methods

### Overview

We conducted a retrospective cohort study of all individuals aged 18 years and older with a recent diagnosis of cancer. Data were extracted from Regenstrief Institute’s Indiana Network for Patient Care (INPC). The INPC [27], developed almost 30 years ago, is among the largest regional health information exchange (HIE) networks and contains over 17 million patient-level medical records. Medical records served as the source for identifying cancer diagnoses using the International Classification of Diseases, 10th Revision codes in the population. Moreover, due to its role in supporting population-level surveillance during the COVID-19 pandemic [28,29], the INPC medical records are linked to governmental, hospital, and private SARS-CoV-2 testing data from across the state. The integrated data set provided comprehensive information on cancer diagnosis along with SARS-CoV-2 infection during the entire study period. This includes information on positive SARS-CoV-2 individuals with cancer who did not present for medical care (eg, asymptomatic individuals and individuals with mild symptoms). The INPC further contains information from death certificates (eg, date of death) provided by the Indiana Department of Health. This enabled determination of death status among individuals who were not treated for COVID-19 in a hospital setting.

All individuals 18 years and older, with an incident of cancer diagnosis between January 1, 2019, and December 31, 2020, were included for this study. Individuals with a prior history of cancer (eg, diagnosis before January 1, 2019) were excluded. Because INPC diagnoses date back to 2011 for most institutions, the data source possessed sufficient documentation of prior cancer diagnosis and treatment. In keeping with practice guidelines, individuals were deemed to have COVID-19 if a reverse transcription polymerase chain reaction assay test from a throat or nose swab was positive for the SARS-CoV-2 virus. Participants with a clinical or radiological diagnosis of COVID-19 without the reverse transcription polymerase chain reaction test were not included in this study. This report is based on the Strengthening the Reporting of Observational Studies in Epidemiology (STROBE) guidelines for cohort studies [30].

### Covariates

Covariates for the study included demographic and clinical variables obtained from the INPC. The demographic variables were age groups in years (18-44, 45-64, and ≥65), sex, race or ethnicity (White, Black, Hispanic, and other), and region of residence (rural or urban). The region of residence was defined based on the Rural-Urban Commuting Area codes [31]. The clinical variables were number of chronic diseases, cancer types, and staging. The number of chronic disease variable was categorized as (“0,” “1,” and “≥2”), and cancer types were classified according to International Classification of Diseases, 10th Revision codes (Table S5 in Multimedia Appendix 1). The “Other” category included cancers of the bone and articular tissue, endocrine glands, central nervous system, mesothelioma and soft tissue, male reproductive organs (excluding prostate), and female reproductive organs. The “Other digestive” cancer group included esophagus, stomach, small intestine, liver and intrahepatic bile ducts, gall bladder, and pancreas. Individuals with nonmelanoma skin cancers were excluded from the study [17,18]. The list of chronic diseases, obtained from prior studies [10,17,18], included hypertension, diabetes, coronary heart disease, chronic kidney disease, cerebrovascular disease, hepatitis, and chronic obstructive pulmonary disease.

Staging information was captured using natural language processing from free text within the clinical notes stored in the INPC. We used nDepth, a natural language processing tool developed at the Regenstrief Institute to extract tumor-node-metastasis concepts from oncology notes provided by the comprehensive cancer centers in Indiana to the INPC. The work was developed from previous research and validated in earlies studies [32-35]. The cancer-staging variable was classified as I, II, III, and IV based on the tumor-node-metastasis classification [36].

### Exposure

We included time from cancer diagnosis to SARS-CoV-2 infection as a time-dependent variable as the first exposure variable. We further divided time as a binary variable defined as COVID-19 period, where the pre–COVID-19 (January 1, 2019, to January 14, 2020) period was coded as 0 and the COVID-19 period (January 15, 2020, to December 31, 2020) was coded as 1.

### Outcome

The primary endpoint was all-cause mortality, which was evaluated between January 1, 2019, and December 31, 2020. All individuals who were alive on December 31, 2020, were considered as right-censored observations in this analysis.

### Ethical Considerations

Study approval was obtained from the Indiana University Institutional Review Board (Exempt Protocol #2009667926). Informed consent was waived due to retrospective use of preexisting, deidentified data from medical records.

### Statistical Analysis

**Figure 1:**
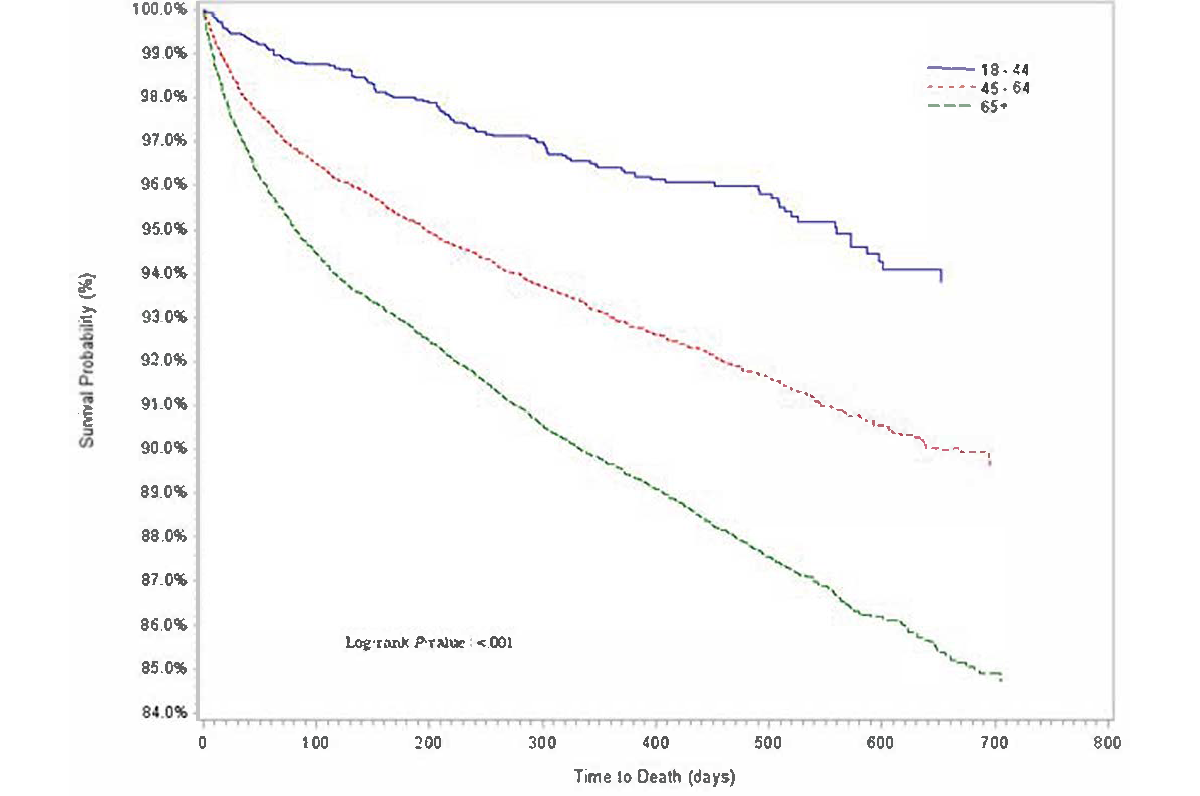
Kaplan-Meier survival estimates of overall cancer population by age group (years), Indiana, 2019-2020.

**Figure 2:**
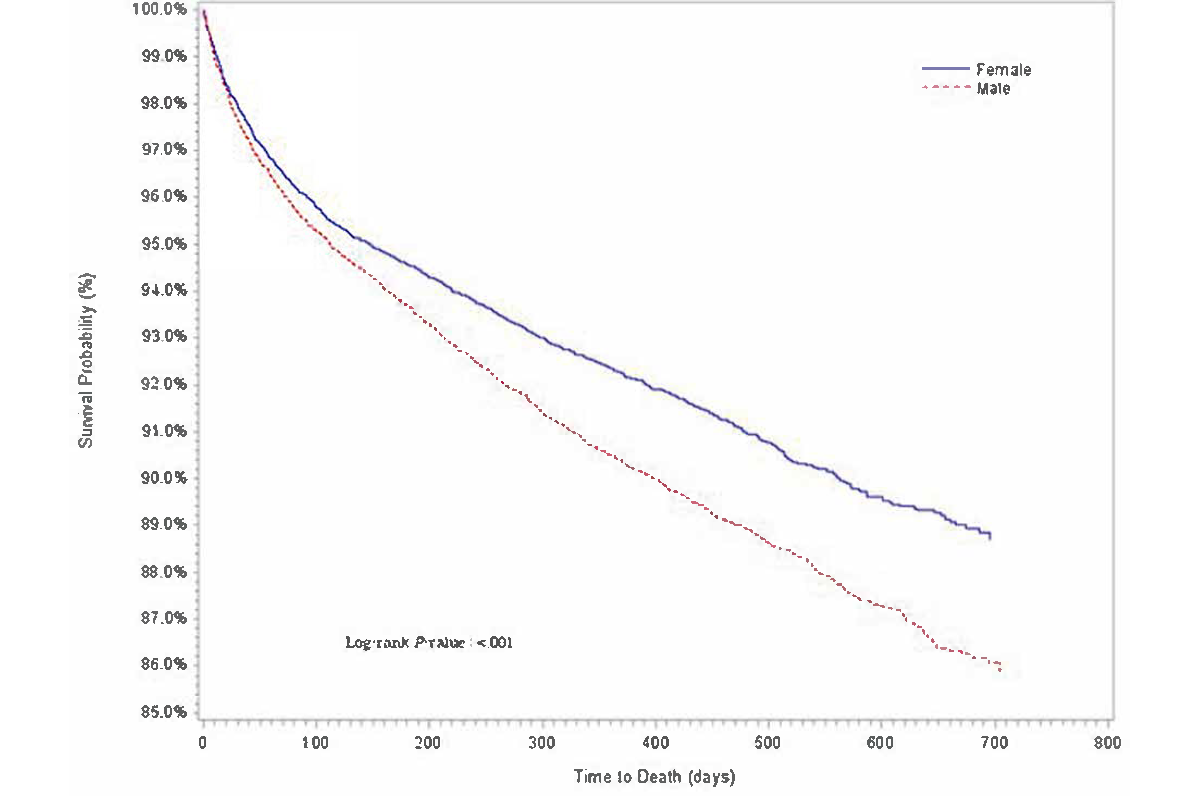
Kaplan-Meier survival estimates of overall cancer population by sex, Indiana, 2019-2020.

**Figure 3:**
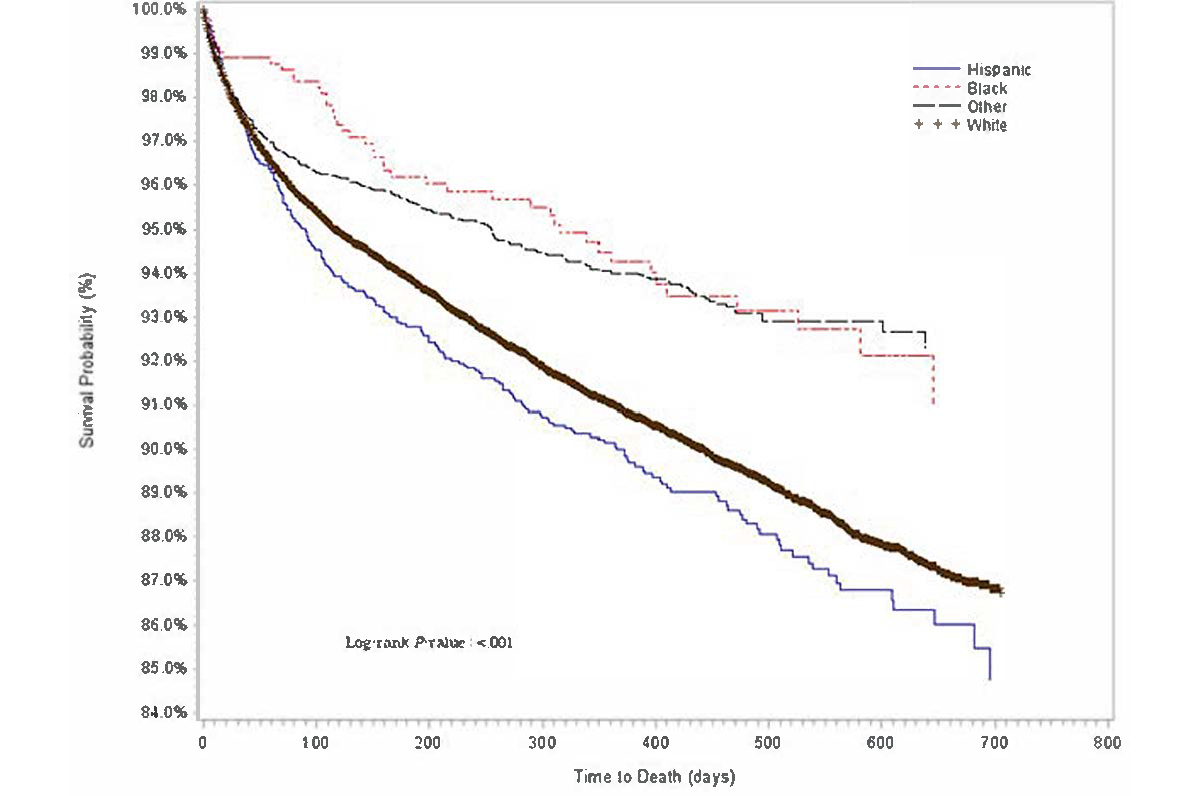
Kaplan-Meier survival estimates of overall cancer population by race and ethnicity, Indiana, 2019-2020.

**Figure 4:**
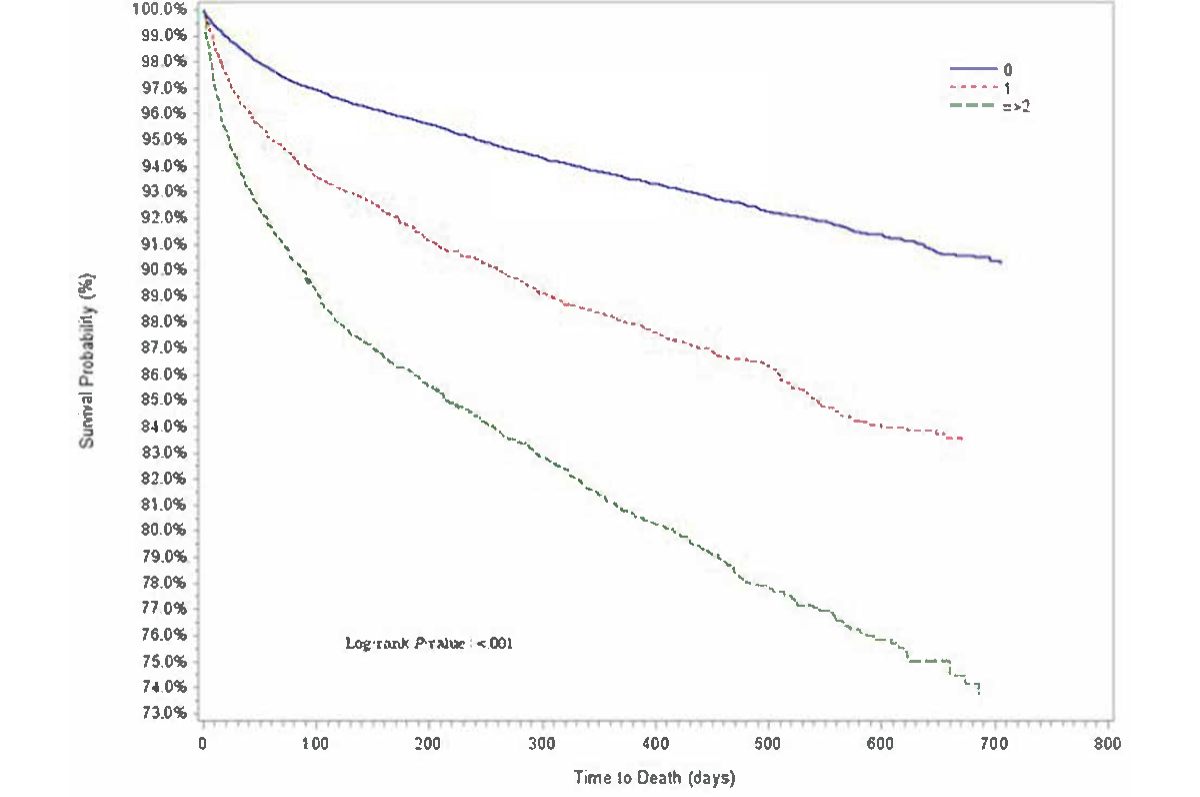
Kaplan-Meier survival estimates of overall cancer population by number of comorbidities, Indiana, 2019-2020.

**Figure 5:**
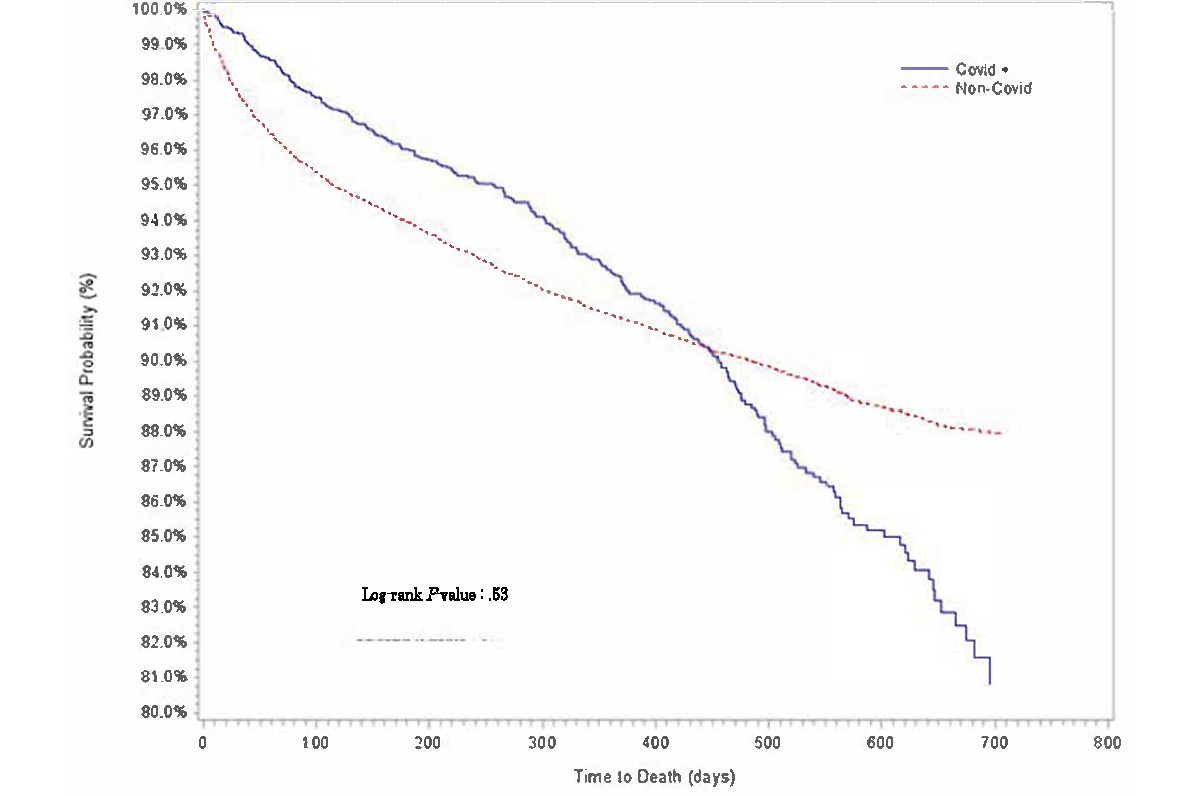
Kaplan-Meier survival estimates of overall cancer population by SARS-CoV-2 diagnosis, Indiana, 2019-2020.

Descriptive statistics of baseline demographic and clinical characteristics are presented in Table 1. The distributions for cumulative proportion of survival over time for age group, sex, race or ethnicity, comorbidities, and COVID-19 diagnosis were estimated using Kaplan-Meier method (Figures 1-5).

The study period started from January 1, 2019, and follow-up ended at death or at the end of the study period on December 31, 2020. In this approach, we compared mortality during the COVID-19 period (January 15, 2020, to December 31, 2020) with that of the pre–COVID-19 period (January 1, 2019, to January 14, 2020). We allowed for pre–COVID-19 period follow-up of survivors to be censored on January 14, 2020, accounting for comparable average time to each event in each period. First, we estimated the survival function using the nonparametric Kaplan-Meier estimator for both the COVID-19 and the pre–COVID-19 period. Next, we estimated the effect of the COVID-19 period variable on all-cause mortality using a Cox proportional hazards model that adjusted for other demographic and clinical variables.

We assessed SARS-CoV-2 infection as a time-dependent exposure. We allocated time spent before a positive SARS-CoV-2 laboratory test to the group that did not have COVID-19 and time spent after the first positive SARS-CoV-2 laboratory test to the group that were confirmed to have COVID-19. This time-dependent approach reduces the immortal-time bias [37]. We estimated mortality hazard ratios (HRs) using time-dependent Cox proportional hazards models [38] adjusting for relevant covariates and assessing proportionality [39] assumptions with cumulative martingale residuals and the Supremum test.

**Table 1:** Characteristics of study cohort diagnosed with cancer during the pre–COVID-19 (January 1, 2019, to January 14, 2020) and COVID-19 (January 15, 2020, to December 31, 2020) periods—Indiana, 2019-2020 (N=41,924).

Characteristics			Pre–COVID-19 period				COVID-19 period					Total
			Dead				Dead					
			No (n=18,895), n (%)		Yes (n=2074), n (%)		No (n=19,631), n (%)		Yes (n=1324), n (%)			
**Age range (years)**												
	18-44	1638 (8.7)		86 (4.2)		1635 (8.3)		30 (2.3)		3389 (8.1)		
	45-64	7005 (37.0)		631 (30.4)		7100 (36.2)		366 (27.6)		15,102 (36.0)		
	≥65	10,252 (88.3)		1357 (65.4)		10,896 (55.5)		928 (70.1)		23,433 (55.9)		
**Race or ethnicity**												
	White	15,051 (79.6)		1694 (81.7)		15,015 (76.5)		1107 (83.6)		32,867 (78.4)		
	Black	1239 (6.6)		166 (8.0)		1173 (6.0)		93 (7.0)		2671 (6.4)		
	Hispanic	416 (2.2)		28 (1.3)		371 (1.9)		17 (1.3)		832 (2.0)		
	Other	2189 (11.6)		186 (9.0)		3072 (15.6)		107 (8.1)		5554 (13.2)		
**Sex**												
	Female	9345 (49.5)		889 (42.9)		9725 (49.5)		611 (46.1)		20,570 (49.1)		
	Male	9550 (50.5)		1185 (57.1)		9906 (50.5)		713 (53.9)		21,354 (50.9)		
**Comorbidities**												
	0	13,863 (73.4)		1095 (52.8)		14,567 (74.2)		699 (52.8)		30,224 (72.1)		
	1	3221 (17.0)		495 (23.9)		3313 (16.9)		324 (24.5)		7353 (17.5)		
	≥2	1811 (9.6)		484 (23.3)		1751 (8.9)		301 (22.7)		4347 (10.4)		
**Cancer**												
	Breast	2743 (14.5)		101 (4.9)		2919 (14.9)		34 (2.6)		5797 (13.8)		
	Colorectal	1391 (7.4)		131 (6.3)		1438 (7.3)		83 (6.3)		3043 (7.3)		
	Leukemia	665 (3.5)		95 (4.6)		714 (3.6)		65 (4.9)		1539 (3.7)		
	Lip, oral cavity, and pharynx	415 (2.2)		48 (2.3)		466 (2.4)		25 (1.9)		954 (2.3)		
	Lung, trachea, and bronchus	1629 (8.6)		431 (20.8)		1662 (8.5)		322 (24.3)		4044 (9.6)		
	Lymphoma	821 (4.3)		100 (4.8)		859 (4.4)		51 (3.8)		1831 (4.4)		
	Myeloma	411 (2.2)		46 (2.2)		328 (1.7)		26 (2.0)		811 (1.9)		
	Other hematological	97 (0.5)		7 (0.3)		89 (0.5)		2 (0.1)		195 (0.5)		
	Other digestive	1033 (5.5)		367 (17.7)		1139 (5.8)		225 (17.0)		2764 (6.6)		
	Prostate	2592 (13.7)		103 (5.0)		2729 (13.9)		44 (3.3)		5468 (13.0)		
	Skin (melanoma)	2734 (14.5)		98 (4.7)		2511 (12.8)		43 (3.2)		5386 (12.8)		
	Urinary tract	1270 (6.7)		125 (6.0)		1399 (7.1)		78 (5.9)		2872 (6.8)		
	Other^a^	3094 (16.4)		422 (20.3)		3378 (17.2)		326 (24.6)		7220 (17.2)		
**COVID-19 positive**												
	No	17,545 (92.9)		1942 (93.6)		18,346 (93.4)		1197 (90.4)		39,030 (93.1)		
	Yes	1350 (7.1)		132 (6.4)		1285 (6.5)		127 (9.6)		2894 (6.9)		
**Region**												
	Urban	14,436 (76.4)		1536 (74.1)		14,222 (72.5)		952 (71.9)		31,146 (74.3)		
	Rural	4459 (23.6)		538 (25.9)		5409 (27.5)		372 (28.1)		10,778 (25.7)		

^a^Includes cancer types such as malignant neoplasia of the bone and articular tissue, endocrine glands, central nervous system, mesothelioma and soft tissue, male reproductive organs (excluding prostate), and female reproductive organs.

### Sensitivity Analysis

We further performed a sensitivity analysis with the 2 methods (time-dependent and COVID-19 period analysis) by additionally adjusting for cancer staging. The missingness of the staging variable was considered “missing not at random”; therefore, we performed the analysis on individuals who had the information on the cancer-staging variable. Baseline characteristics are found in Table S1 in Multimedia Appendix 1, COVID-19 period analysis is presented in Table S2 in Multimedia Appendix 1, and the time-dependent analysis from cancer to COVID-19 diagnosis is presented in Table S3 in Multimedia Appendix 1.

An additional time-dependent analysis was performed on individuals restricted to incident cancer cases from January 15, 2020, to December 31, 2020, to further reduce a possible immortal-time bias (Table S4 in Multimedia Appendix 1). All comparisons were 2-sided, and *P*<.05 was considered statistically significant. Analyses were conducted with SAS version 9.4 (SAS Institute).

## Results

### Cohort Characteristics

The study population consisted of 41,924 individuals with an incident diagnosis of cancer between January 1, 2019, and December 31, 2020 (Table 1). Of these, 2894 (6.2%) were laboratory-confirmed SARS-CoV-2 cases. Most cancer patients were white (n=32,867, 78.4%) and male (n=21,354, 50.9%). Approximately 1 in 5 (n=3221, 17.0%) patients with cancer possessed a single comorbid condition, and 1 in 10 (n=1811, 9.6%) had ≥2 comorbid conditions at the time of cancer diagnosis. Most patients were diagnosed with breast (n=5797, 13.8%), prostate (n=5468, 13.0%), and melanoma (n=5386, 12.8%) cancers during the study period.

### All-Cause Mortality

During the study period, 3398 (8.1%) individuals died. In the COVID-19 period, there were 1324 (38.9%) deaths. Of the individuals who died in the COVID-19 period, 127 (9.6%) tested positive for SARS-CoV-2 (Table 1).

### COVID-19 Period Analysis

The COVID-19 period analysis (Table 2) was adjusted for age group, sex, race or ethnicity, number of comorbidities, cancer type, and region of residence. Mortality was 91% higher during the COVID-19 period (adjusted HR 1.91; 95% CI 1.76-2.09; *P*<.001) compared to the pre–COVID-19 period. The risk of mortality was 3-fold higher among adults 65 years and older (adjusted HR 3.35; 95% CI 2.58-4.35; *P*<.001) compared to adults in the age group of 18-44 years. Risk of mortality among adults in rural residence (adjusted HR 0.99; 95% CI 0.91-1.09; *P*=.87) was 1% lower compared to urban residence, but it was not statistically significant.

**Table 2:** Unadjusted and adjusted Cox-regression analyses using landmarks during the pre–COVID-19 and COVID-19 periods as well as all-cause mortality—Indiana, 2019-2020 (N=41,924).

Variable			Unadjusted						Adjusted					
			Estimate (SE)		Hazard ratio (95% CI)		*P* value		Estimate (SE)		Hazard ratio (95% CI)		*P* value	
**Covid-19 period^a^**														
	No	N/A^b^		Reference		<.001		N/A		Reference		<.001		
	Yes	0.63 (0.04)		1.88 (1.73-2.05)				0.65 (0.04)		1.91 (1.76-2.09)				
**Age group (years)**														
	18-44	N/A		Reference		<.001		N/A		Reference		<.001		
	45-64	0.91 (0.13)		2.48 (1.91-3.22)				0.86 (0.13)		2.36 (1.81-3.08)				
	≥65	1.33 (0.13)		3.80 (2.94-4.91)				1.20 (0.13)		3.35 (2.58-4.35)				
**Sex**														
	Female	N/A		Reference		<.001		N/A		Reference		<.001		
	Male	0.16 (0.04)		1.17 (1.08-1.27)				0.18 (0.04)		1.20 (1.10-1.30)				
**Race or ethnicity**														
	White	N/A		Reference		<.001		N/A		Reference		<.001		
	Black	0.13 (0.08)		1.14 (0.98-1.34)				0.14 (0.08)		1.15 (0.98-1.34)				
	Hispanic	–0.74 (0.21)		0.48 (0.32-0.72)				–0.60 (0.21)		0.55 (0.36-0.83)				
	Other	–0.27 (0.07)		0.76 (0.66-0.87)				–0.20 (0.07)		0.82 (0.71-0.94)				
**Comorbidities**														
	0	N/A		Reference		<.001		N/A		Reference		<.001		
	1	0.66 (0.05)		1.94 (1.76-2.15)				0.39 (0.05)		1.48 (1.33-1.63)				
	≥2	1.20 (0.05)		3.31 (2.99-3.67)				0.82 (0.05)		2.27 (2.04-2.52)				
**Cancer**														
	Other	N/A		Reference		<.001		N/A		Reference		<.001		
	Breast	–1.82 (0.12)		0.16 (0.13-0.21)				–1.72 (0.13)		0.17 (0.14-0.23)				
	Colorectal	–0.51 (0.09)		0.60 (0.50-0.72)				–0.61 (0.09)		0.54 (0.45-0.66)				
	Leukemia	0.08 (0.09)		1.08 (0.89-1.32)				–0.03 (0.10)		0.96 (0.79-1.17)				
	Lip, oral cavity, and pharynx	–0.52 (0.16)		0.59 (0.44-0.81)				–0.60 (0.16)		0.55 (0.40-0.75)				
	Lung and bronchus	0.59 (0.06)		1.82 (1.61-2.04)				0.33 (0.06)		1.39 (1.23-1.57)				
	Lymphoma	–0.28 (0.11)		0.76 (0.61-0.93)				–0.29 (0.11)		0.74 (0.60-0.92)				
	Myeloma	–0.34 (0.15)		0.71 (0.52-0.96)				–0.53 (0.15)		0.58 (0.43-0.80)				
	Other digestive	0.72 (0.06)		2.07 (1.82-2.35)				0.53 (0.06)		1.69 (1.48-1.93)				
	Other hematological	–1.63 (0.33)		0.41 (0.21-0.80)				–1.75 (0.58)		0.17 (0.06-0.54)				
	Prostate	–1.70 (0.12)		0.25 (0.21-0.30)				–1.94 (0.12)		0.14 (0.11-0.18)				
	Melanoma	–1.83 (0.13)		0.24 (0.20-0.28)				–1.93 (0.13)		0.14 (0.11-0.19)				
	Urinary tract	–0.52 (0.09)		0.67 (0.57-0.78)				–0.79 (0.09)		0.45 (0.37-0.55)				
**Region**														
	Urban	N/A		Reference		<.001		N/A		Reference		<.001		
	Rural	0.07 (0.04)		1.07 (0.98-1.17)				0.007 (0.05)		0.99 (0.91-1.09)				

^a^COVID-19 period calculated as a binary variable; no=January 1, 2019, to January 14, 2020; yes=January 15, 2020, to December 31, 2020.

^b^N/A: not applicable.

### Time-Dependent Analysis

After adjusting for age, race, sex, number of comorbidities, and cancer subtypes, SARS-CoV-2 infection was associated with a 7-fold increase in the hazard of death (adjusted HR 6.91; 95% CI 6.06-7.89; *P*<.001; Table 3). The hazard of death was 23% higher for male individuals (adjusted HR 1.23; 95% CI 1.14-1.32; *P*<.001), compared to female individuals. All-cause mortality was higher by 45% in the lung cancer group (adjusted HR 1.45; 95% CI 1.31-1.61; *P*<.001) and 80% in other digestive cancers group (adjusted HR 1.80; 95% CI 1.61-2.00; *P*<.001), compared to other cancer types.

**Table 3:** Unadjusted and adjusted time-dependent (cancer diagnosis to COVID-19 diagnosis) Cox-regression analysis and all-cause mortality—Indiana, 2019-2020.

Variable		Unadjusted				Adjusted			
		Estimate (SE)	Hazard ratio (95% CI)	*P* value	Estimate (SE)		Hazard ratio (95% CI)	*P* value	
**Covid-19 diagnosis**									
	No	N/A^a^	Reference	<.001	N/A		Reference	<.001	
	Yes	1.87 (0.07)	6.53 (5.72-7.44)		1.93 (0.07)		6.91 (6.06-7.89)		
**Age group (years)**									
	8-44	N/A	Reference	<.001	N/A		Reference		
	45-64	0.69 (0.09)	2.00 (1.65-2.42)		0.65 (0.09)		1.91 (1.57-2.32)		
	≥65	1.20 (0.09)	3.10 (2.54-3.70)		1.01 (0.09)		2.74 (2.26-3.31)		
**Sex**									
	Female	N/A	Reference	<.001	N/A		Reference	<.001	
	Male	0.19 (0.03)	1.21 (1.13-1.30)		0.21 (0.04)		1.23 (1.14-1.32)		
**Race or ethnicity**									
	White	N/A	Reference	<.001	N/A		Reference	<.001	
	Black	0.11 (0.06)	1.12 (0.98-1.27)		0.10 (0.06)		1.11 (0.97-1.26)		
	Hispanic	–0.50 (0.15)	0.60 (0.45-0.81)		–0.47 (0.15)		0.62 (0.46-0.84)		
	Other	–0.34 (0.06)	0.71 (0.63-0.80)		–0.23 (0.06)		0.79 (0.70-0.90)		
**Comorbidities**									
	0	N/A	Reference	<.001	N/A		Reference	<.001	
	1	0.65 (0.04)	1.91 (1.75-2.07)		0.37 (0.04)		1.44 (1.33-1.57)		
	≥2	1.15 (0.04)	3.16 (2.90-3.43)		0.75 (0.04)		2.12 (1.94-2.31)		
**Cancer**									
	Other	N/A	Reference	<.001	N/A		Reference	<.001	
	Breast	–1.51 (0.09)	0.22 (0.18-0.26)		–1.43 (0.09)		0.24 (0.20-0.29)		
	Colorectal	–0.41 (0.07)	0.67 (0.57-0.78)		–0.51 (0.07)		0.60 (0.51-0.70)		
	Leukemia	0.03 (0.08)	1.03 (0.87-1.22)		–0.10 (0.09)		0.90 (0.76-1.07)		
	Lip, oral cavity, and pharynx	–0.31 (0.12)	0.73 (0.58-0.93)		–0.39 (0.12)		0.67 (0.53-0.86)		
	Lung and bronchus	0.63 (0.05)	1.87 (1.69-2.07)		0.38 (0.05)		1.45 (1.31-1.61)		
	Lymphoma	–0.25 (0.09)	0.79 (0.66-0.94)		–0.31 (0.09)		0.73 (0.61-0.87)		
	Myeloma	–0.24 (0.12)	0.78 (0.61-0.99)		–0.44 (0.12)		0.64 (0.50-0.82)		
	Other digestive	0.78 (0.05)	2.18 (1.96-2.43)		0.58 (0.05)		1.80 (1.61-2.00)		
	Other hematological	–0.90 (0.33)	0.41 (0.21-0.78)		–1.06 (0.33)		0.35 (0.18-0.67)		
	Prostate	–1.38 (0.09)	0.25 (0.21-0.30)		–1.64 (0.09)		0.19 (0.16-0.23)		
	Melanoma	–1.43 (0.09)	0.24 (0.20-0.28)		–1.56 (0.09)		0.21 (0.17-0.25)		
	Urinary tract	–0.39 (0.08)	0.67 (0.58-0.79)		–0.68 (0.08)		0.50 (0.43-0.59)		
**Region**									
	Urban	N/A	Reference	<.001	N/A		Reference	<.001	
	Rural	0.13 (0.04)	1.14 (1.06-1.23)		0.08 (0.04)		1.09 (1.01-1.18)		

^a^N/A: not applicable.

### Sensitivity Analysis

Finally, we assessed the impact of cancer-staging variable (n=9567) in a sensitivity analysis (Table S1 in Multimedia Appendix 1). Risk of death increased over 2.5-fold after SARS-CoV-2 infection (adjusted HR 2.55; 95% CI 2.17-2.99; *P*<.001) after adjusting for other covariates and the staging variable (Table S2 in Multimedia Appendix 1) in the COVID-19 period. In the time-dependent analysis, the hazard of death increased by 4.5-fold (adjusted HR 4.63; 95% CI 3.58-5.99) in the COVID-19 period compared to the pre–COVID-19 period, after adjusting for the other covariates and the staging variable (Table S3 in Multimedia Appendix 1).

## Discussion

### Principal Findings

In this large population-based study of individuals with recently diagnosed cancer, we found that SARS-CoV-2 infection negatively impacted survival with a 7-fold increase in death. Survival was largely impacted among adults with increasing age, those with 2 or more comorbid conditions, and among males. Individuals with lung cancer and other digestive cancers had the highest risk of death after SARS-CoV-2 infection. This analysis provides additional empirical evidence on the magnitude of risk to patients with cancer whose immune systems are often weakened either by the disease or treatment. This evidence may help providers and public health authorities take steps to improve outcomes among people with cancer, including messaging for vaccination campaigns as well as the need for continued vigilance against infection given continued waves of infection in the United States, Europe, and other nations.

### Comparison With Prior Work

One of the notable findings from this study has been that SARS-CoV-2 infection shows a markedly increased risk of death in comparison to previous studies [10,11,14,15,17,40,41]. Previous case-control studies from China reported findings of 2-fold [10] and 3-fold [11] increases in all-cause mortality, which are much lower compared to our finding of a 7-fold increase in the time-dependent analysis. Multicenter cohort studies from Europe [14,18,40,41] and the United States [4,15,42] have reported similar findings on subgroups such as age, sex, and race. In retrospect, our statewide findings support the important policy decisions that were undertaken at the height of the pandemic of limiting exposure of SARS-CoV-2 for the general population and for individuals with cancer and other immunocompromised populations. However, some of these measures diverted resources to control the spread of the virus and reduced cancer screening access with a short drop in cancer diagnosis [43]. In comparison to the general population, our prevalence of SARS-CoV-2 (n=2894, 6.2%) in this study was substantially lower compared to the general population (17.9%) [44] in Indiana. Thus, showing that the cancer population likely took preventive measures to avoid exposure to the SARS-CoV-2 virus.

Our finding of increased mortality in individuals with lung cancer and other digestive cancers can partly explain the increased mortality among males. This increased mortality for lung cancers could be related to the underlying disease (eg, bronchial carcinoma) or due to coronaviruses that cause severe lung injury [45] observed during the SARS-CoV-1 outbreak in 2003 [46]. It may also be related to underlying health behaviors, including smoking [47], which is also associated with lung cancer. We found significantly lower mortality among hematological malignancies compared to the other solid tumors such as lung, digestive, and breast cancer. This contrasts with a multicenter study [14] in Europe and the United States, which found 40% increased odds in mortality for hematological cancers (such as lymphoma, leukemia, and myeloma) compared to solid tumors. This rather contradictory result may be explained by the fact that the prior study [14] grouped the cancers into three broader categories such as hematological, solid, and multiple cancers, which differ from our cancer variable that includes individual cancer types. Our population-based analysis enabled sufficient power for more granular analysis of individual cancer types compared with prior work.

In comparison to similar studies [4,14-16,18] of cancer cohorts, we accounted for time, which better explains the increased hazard due to SARS-CoV-2 infection. Thus, the increased hazard from our study highlights the need to further continue the care planning and conversations with family on treatment options, vaccinations, and SARS CoV-2 testing during treatment [14,15]. Recent findings demonstrate that individuals with compromised immune systems, including patients with cancer, do not have a strong protection from COVID-19 after full vaccination (eg, 2 doses of mRNA vaccine) [24-26]. Therefore, both the US Centers for Disease Control and Prevention and the US Food and Drug Administration recommend patients who have cancer and are receiving mRNA COVID-19 vaccines should receive 3 doses and a booster, practice nonpharmaceutical interventions, and, if infected, be monitored closely and considered early for proven therapies that can prevent severe outcomes [25,48].

The strengths of the study include that we were able to account for time to diagnosis of SARS-CoV-2 infection, which was a limitation for prior studies due to the nature of a global crisis. Second, we were able to account for individuals with cancer and without COVID-19, which places our findings in better context compared with previous studies that reported findings only among individuals with cancer and a COVID-19 diagnosis. Third, we were able to leverage data from a large statewide HIE representative of the underlying population, which supports generalization of findings to cancer populations compared to single institutions or multicenter studies in more narrow geographic regions. For example, one-third of Indiana’s population lives in rural areas, and the HIE accesses electronic health records from community hospitals in addition to 3 large regional cancer care providers in the state. Therefore, our findings strengthen the justification of treatment planning and care guidelines required for the management of patients with cancer, which were highlighted in prior studies.

### Limitations

There are some limitations in our study. First, our analysis is dependent on the testing and reporting of SARS-CoV-2 from different facilities. This could underreport total COVID-19 cases in patients with cancer; however, this bias would be only toward those individuals who never received testing, given the HIE is linked to statewide testing databases. Early in the pandemic, however, testing was limited to symptomatic individuals. This could have led to testing among those with only severe COVID-19, thereby overestimating severity. Second, we were unable to account for COVID-19 severity and treatment confounders in our study; however, from previous studies, we know that treating the underlying cancer and with regular testing were prudent choices in either initiating or continuing with cancer treatment. In addition, cancer diagnoses [49-53] were ascertained via diagnostic codes, which introduces known measurement errors in terms of positive and negative predictive value. Lastly, the impact of the pandemic led to a substantial reduction in new cancer diagnosis and health services across the US [43,54] and globally [55-57], which might have overestimated the HR for our study.

### Conclusion

In summary, this large study among individuals with cancer and SARS-CoV-2 emphasizes the need for continuing testing and monitoring patients for the delivery of oncology services. Given the risk of severe disease and mortality is substantially larger for patients who have cancer, the study further underscores the need for patients with cancer to receive complete vaccination, including recommended booster doses authorized by the Food and Drug Administration as well as Centers for Disease Control and Prevention.

## Appendix

Multimedia Appendix 1 Supplemental tables.

## Abbreviations

HIE: health information exchange
HR: hazard ratio
INPC: Indiana Network for Patient Care
STROBE: Strengthening the Reporting of Observational Studies in Epidemiology
