# Supplementary material for: COVID-19 Diagnosis and Risk of Death Among Adults With Cancer in Indiana: Retrospective Cohort Study
Source: JMIR Cancer. 2022 Oct 6;8(4):e35310. doi: 10.2196/35310 (PMC9555821; doi:10.2196/35310)
Supplement: Multimedia Appendix 1 [file cancer_v8i4e35310_app1.docx]

| **TABLE S1. Demographic characteristics of individuals diagnosed with cancer during the pre-COVID-19 (January 1, 2019 to January 14, 2020) and COVID-19 period (January 15, 2020 to December 31, 2020) – Indiana, 2019-2020.** | | | | | |
| --- | --- | --- | --- | --- | --- |
|  | **Pre-COVID-19** | | **COVID-19 period** | | **Total** |
|  | **Dead** | | **Dead** | |  |
| **Characteristics** | **No** | **Yes** | **No** | **Yes** |  |
|  | n = 5234 | n = 299 | n = 3671 | n = 363 | 9567 |
| **Age group, y** |  |  |  |  |  |
| 18—44 | 515 (9.8) | 6 (2.0) | 370 (10.1) | 11 (3.0) | 902 (9.4) |
| 45—64 | 1992 (38.0) | 102 (34.1) | 1499 (40.8) | 119 (32.8) | 3712 (38.8) |
| ≥65 | 2727 (5.2) | 191 (63.9) | 1802 (49.1) | 233 (64.2) | 4953 (51.8) |
| **Race** |  |  |  |  |  |
| White | 4545 (86.8) | 249 (83.3) | 3204 (87.3) | 318 (87.6) | 8316 (86.9) |
| Black | 407 (7.8) | 24 (8.3) | 276 (7.5) | 30 (8.3) | 737 (7.7) |
| Hispanic | 111 (2.1) | 0 (0.0) | 78 (2.1) | 5 (1.4) | 194 (2.0) |
| Other | 171 (3.3) | 26 (8.7) | 113 (3.1) | 10 (2.7) | 320 (3.4) |
| **Sex** |  |  |  |  |  |
| Female | 2851 (54.5) | 125 (41.8) | 2098 (57.2) | 170 (46.8) | 5244 (54.8) |
| Male | 2383 (45.5) | 174 (58.2) | 1573 (42.8) | 193 (53.2) | 4323 (45.2) |
| **Comorbidities** |  |  |  |  |  |
| 0 | 3388 (64.7) | 151 (50.5) | 2443 (66.5) | 170 (46.8) | 6152 (64.3) |
| 1 | 1068 (20.4) | 69 (23.1) | 719 (19.6) | 87 (24.0) | 1943 (20.3) |
| ≥2 | 778 (14.9) | 79 (26.4) | 509 () | 106 (29.2) | 1472 (15.4) |
| **Cancer** |  |  |  |  |  |
| Breast | 927 (17.8) | 4 (1.3) | 741 (20.2) | 7 (1.9) | 1679 (17.5) |
| Colorectal | 475 (9.1) | 13 (4.3) | 361 (9.8) | 22 (6.1) | 871 (9.1) |
| Leukemia | 126 (2.4) | 14 (4.7) | 81 (2.2) | 10 (2.7) | 231 (2.4) |
| Lip, oral cavity & pharynx | 155 (3.0) | 7 (2.3) | 143 (3.9) | 9 (2.5) | 314 (3.3) |
| Lung, trachea & bronchus | 645 (12.3) | 86 (28.8) | 411 (11.2) | 92 (25.3) | 1234 (12.9) |
| Lymphoma | 270 (5.2) | 20 (6.7) | 205 (5.6) | 13 (3.6) | 508 (5.3) |
| Myeloma | 142 (2.7) | 5 (1.7) | 101 (2.7) | 101 (27.8) | 253 (2.6) |
| Other hematological | 21 (0.4) | 0 (0.0) | 17 (0.5) | 0 (0.0) | 38 (0.4) |
| Other digestive | 467 (8.9) | 68 (22.7) | 324 (8.8) | 82 (22.6) | 941 (9.8) |
| Prostate | 368 (7.0) | 9 (3.0) | 236 (6.4) | 12 (3.3) | 625 (6.5) |
| Skin (Melanoma) | 418 (8.0) | 6 (2.0) | 214 (5.8) | 10 (2.7) | 648 (6.8) |
| Urinary tract | 234 (4.5) | 10 (3.3) | 117 (3.2) | 14 (3.8) | 375 (3.9) |
| Other | 986 (18.8) | 57 (19.1) | 720 (19.6) | 87 (24.0) | 1850 (19.3) |
| **Cancer stage** |  |  |  |  |  |
| 1 | 1605 (32.7) | 103 (12.2) | 1228 (32.0) | 31 (8.1) | 2967 (29.8) |
| 2 | 985 (20.1) | 131 (15.6) | 749 (19.5) | 33 (8.6) | 1898 (19.0) |
| 3 | 1288 (26.2) | 206 (24.5) | 1033 (27.0) | 120 (31.4) | 2647 (26.5) |
| 4 | 1033 (21.0) | 402 (47.7) | 823 (21.5) | 198 (51.8) | 2456 (24.6) |
| **COVID-19 positive** |  |  |  |  |  |
| No | 4865 (92.9) | 299 (100.0) | 3397 (92.5) | 336 (92.6) | 8897 (93.0) |
| Yes | 369 (7.1) | 0 (0.0) | 274 (7.5) | 27 (7.4) | 670 (7.0) |
| **Region** |  |  |  |  |  |
| Urban | 4128 (78.9) | 226 (75.6) | 2904 (79.1) | 305 (84.0) | 7563 (79.0) |
| Rural | 1106 (21.1) | 73 (24.4) | 767 (20.9) | 58 (16.0) | 2004 (21.0) |

| **TABLE S2. Unadjusted and adjusted Cox-regression analyses using landmarks pre-COVID-19 and COVID-19 period and all-cause mortality–Indiana, 2019-2020** | | | | | | |
| --- | --- | --- | --- | --- | --- | --- |
|  | **Unadjusted** | | | **Adjusted** | | |
| **Variable** | **Estimate**  **(SE)** | **Hazard ratio (95% CI)** | ***P*** | **Estimate**  **(SE)** | **Hazard ratio (95% CI)** | ***P*** |
| **Covid-period*** |  |  |  |  |  |  |
| No | -- | **Ref** | <.001 | -- | **Ref** | <.001 |
| Yes | 0.92 (0.08) | 2.53 (2.16-2.97) |  | 0.93 (0.08) | 2.55 (2.17-2.99) |  |
| **Age group, y** |  |  |  |  |  |  |
| 18—44 | -- | **Ref** |  | -- | **Ref** | <.001 |
| 45—64 | 1.22 (0.26) | 3.39 (2.04-5.64) |  | 0.91 (0.26) | 2.48 (1.49-4.14) |  |
| ≥65 | 1.56 (0.25) | 4.77 (2.89-7.86) |  | 1.93 (0.26) | 3.30 (1.98-5.48) |  |
| **Sex** |  |  |  |  |  |  |
| Female | -- | **Ref** | <.001 | -- | **Ref** | 0.10 |
| Male | 0.36 (0.08) | 1.43 (1.23-1.67) |  | 0.13 (0.08) | 1.14 (0.97-1.34) |  |
| **Race** |  |  |  |  |  |  |
| White | -- | **Ref** | 0.04 | -- | **Ref** | <.01 |
| Black | 0.10 (0.14) | 1.11 (0.84-1.47) |  | 0.13 (0.14) | 1.14 (0.86-1.52) |  |
| Hispanic | -0.97 (0.44) | 0.38 (0.16-0.91) |  | -0.79 (0.45) | 0.45 (0.19-1.09) |  |
| Other | 0.57 (0.17) | 1.77 (1.26-2.48) |  | 0.49 (0.17) | 1.64 (1.16-2.30) |  |
| **Comorbidities** |  |  |  |  |  |  |
| 0 | -- | **Ref** | <.001 | -- | **Ref** | <.001 |
| 1 | 0.43 (0.09) | 1.54 (1.27-1.87) |  | 0.19 (0.10) | 1.20 (0.99-1.46) |  |
| ≥2 | 0.88 (0.09) | 2.40 (2.01-2.89) |  | 0.65 (0.09) | 1.92 (1.59-2.33) |  |
| **Cancer** |  |  |  |  |  |  |
| Other | -- | **Ref** | <.001 | -- | **Ref** | <.001 |
| Breast | -2.33 (0.31) | 0.10 (0.05-0.18) |  | -1.75 (0.31) | 0.17 (0.09-0.32) |  |
| Colorectal | -0.63 (0.19) | 0.53 (0.37-0.77) |  | -0.69 (0.19) | 0.50 (0.35-0.73) |  |
| Leukemia | 0.52 (0.22) | 1.68 (1.08-2.61) |  | 0.56 (0.22) | 1.76 (1.12-2.75) |  |
| Lip, oral cavity & pharynx | -0.40 (0.26) | 0.67 (0.40-1.12) |  | -0.42 (0.26) | 0.66 (0.39-1.11) |  |
| Lung and bronchus | 0.62 (0.11) | 1.85 (1.48-2.32) |  | 0.30 (0.11) | 1.35 (1.08-1.70) |  |
| Lymphoma | -0.22 (0.20) | 0.80 (0.54-1.18) |  | -0.21 (0.19) | 0.81 (0.55-1.18) |  |
| Myeloma | -0.67 (0.33) | 0.51 (0.27-0.97) |  | -0.90 (0.33) | 0.41 (0.21-0.77) |  |
| Other digestive | 0.74 (0.11) | 2.10 (1.67-2.65) |  | 0.58 (0.12) | 1.78 (1.41-2.25) |  |
| Prostate | -0.88 (0.23) | 0.41 (0.26-0.65) |  | -0.99 (0.24) | 0.37 (0.23-0.59) |  |
| Melanoma | -1.18 (0.26) | 0.31 (0.18-0.51) |  | -1.01 (0.27) | 0.36 (0.21-0.61) |  |
| Urinary tract | -0.21 (0.22) | 0.81 (0.52-1.24) |  | -0.35 (0.22) | 0.70 (0.45-1.09) |  |
| **Cancer** **stage** |  |  |  |  |  |  |
| 1 | -- | **Ref** | <.001 | -- | **Ref** | <.001 |
| 2 | 0.53 (0.17) | 1.70 (1.22-2.37) |  | 0.53 (0.17) | 1.52 (1.08-2.13) |  |
| 3 | 0.99 (0.14) | 2.70 (2.03-3.61) |  | 0.99 (0.15) | 2.00 (1.49-2.68) |  |
| 4 | 1.83 (0.13) | 6.25 (4.79-8.17) |  | 1.46 (0.14) | 4.30 (3.28-5.65) |  |
| **Region** |  |  |  |  |  |  |
| Urban | -- | **Ref** | 0.25 | -- | **Ref** | 0.25 |
| Rural | -0.11 (0.10) | 0.89 (0.73-1.09) |  | -0.11 (0.10) | 0.89 (0.73-1.08) |  |
| *COVID-era calculated as a binary variable; no = January 1, 2019 to January 14, 2020, yes = January 15, 2020 to December 31, 2020  **Note:** Other hematological cancers were excluded from the analyses due to a lack of events | | | | | | |

| **TABLE S3. Unadjusted and adjusted time-dependent (cancer diagnosis to COVID-19 diagnosis) Cox-regression analysis and all-cause mortality–Indiana, 2019-2020** | | | | | | |
| --- | --- | --- | --- | --- | --- | --- |
|  | **Unadjusted** | | | **Adjusted** | | |
| **Variable** | **Estimate (SE)** | **Hazard ratio**  **(95% CI)** | ***P*** | **Estimate (SE)** | **Hazard ratio**  **(95% CI)** | ***P*** |
| **Covid-diagnosis** |  |  |  |  |  |  |
| No | -- | **Ref** | <.001 | -- | **Ref** | <.001 |
| Yes | 1.43 (0.13) | 4.20 (3.25-5.42) |  | 1.53 (0.13) | 4.63 (3.58-5.99) |  |
| **Age group, y** |  |  |  |  |  |  |
| 18—44 | -- | **Ref** |  | -- | **Ref** | <.001 |
| 45—64 | 0.58 (0.15) | 1.79 (1.34-2.39) |  | 0.24 (0.15) | 1.27 (0.95-1.71) |  |
| ≥65 | 0.93 (0.14) | 2.52 (1.91-3.34) |  | 0.55 (0.15) | 1.74 (1.31-2.33) |  |
| **Sex** |  |  |  |  |  |  |
| Female | -- | **Ref** | <.001 | -- | **Ref** | 0.03 |
| Male | 0.36 (0.06) | 1.44 (1.28-1.62) |  | 0.13 (0.06) | 1.14 (1.01-1.29) |  |
| **Race** |  |  |  |  |  |  |
| White | -- | **Ref** | 0.03 | -- | **Ref** | 0.17 |
| Black | 0.12 (0.09) | 1.13 (0.93-1.37) |  | 0.11 (0.11) | 1.11 (0.90-1.38) |  |
| Hispanic | -0.53 (0.26) | 1.08 (0.87-1.33) |  | -0.49 (0.26) | 0.61 (0.37-1.02) |  |
| Other | -0.46 (0.22) | 0.63 (0.41-0.97) |  | -0.10 (0.14) | 1.10 (0.83-1.47) |  |
| **Comorbidities** |  |  |  |  |  |  |
| 0 | -- | **Ref** | <.001 |  | **Ref** | <.001 |
| 1 | 0.48 (0.07) | 1.62 (1.40-1.87) |  | 0.24 (0.07) | 1.27 (1.10-1.47) |  |
| ≥2 | 0.82 (0.07) | 2.26 (1.97-2.60) |  | 0.60 (0.07) | 1.83 (1.58-2.12) |  |
| **Cancer** |  |  |  |  |  |  |
| Other | -- | **Ref** | <.001 | -- | **Ref** | <.001 |
| Breast | -1.62 (0.17) | 0.20 (0.14-0.28) |  | -1.11 (0.18) | 0.39 (0.23-0.47) |  |
| Colorectal | -0.45 (0.13) | 0.64 (0.49-0.83) |  | -0.53 (0.14) | 0.59 (0.45-0.77) |  |
| Leukemia | 0.30 (0.18) | 1.35 (0.94-1.93) |  | 0.24 (0.18) | 1.27 (0.88-1.82) |  |
| Lip, oral cavity & pharynx | -0.24 (0.20) | 0.79 (0.53-1.16) |  | -0.21 (0.20) | 0.82 (0.55-1.21) |  |
| Lung and bronchus | 0.65 (0.08) | 1.92 (1.62-2.29) |  | 0.40 (0.09) | 1.49 (1.25-1.79) |  |
| Lymphoma | -0.33 (0.16) | 0.72 (0.52-0.98) |  | -0.42 (0.16) | 0.65 (0.47-0.90) |  |
| Myeloma | -0.33 (0.21) | 0.71 (0.47-1.09) |  | -0.27 (0.22) | 0.76 (0.50-1.16) |  |
| Other digestive | 0.82 (0.09) | 2.27 (1.90-2.72) |  | 0.68 (0.09) | 1.98 (1.65-2.37) |  |
| Prostate | -0.58 (0.15) | 0.56 (0.41-0.76) |  | -0.69 (0.16) | 0.50 (0.36-0.69) |  |
| Melanoma | -0.78 (0.16) | 0.46 (0.33-0.63) |  | -0.76 (0.17) | 0.47 (0.34-0.65) |  |
| Urinary tract | -0.06 (0.16) | 0.94 (0.69-1.28) |  | -0.28 (0.16) | 0.76 (0.55-1.04) |  |
| **Cancer** **stage** |  |  |  |  |  |  |
| 1 | -- | **Ref** | <.001 | -- | **Ref** | <.001 |
| 2 | 0.64 (0.12) | 1.90 (1.50-2.40) |  | 0.52 (0.12) | 1.69 (1.34-2.14) |  |
| 3 | 0.96 (0.10) | 2.62 (2.13-3.22) |  | 0.72 (0.11) | 2.05 (1.66-2.54) |  |
| 4 | 1.70 (0.09) | 5.48 (4.52-6.66) |  | 1.40 (0.10) | 4.08 (3.35-4.98) |  |
| **Region** |  |  |  |  |  |  |
| Urban | -- | **Ref** | 0.82 | -- | **Ref** | 0.51 |
| Rural | -0.02 (0.07) | 0.98 (0.85-1.13) |  | -0.05 (0.07) | 0.95 (0.82-1.10) |  |

| **TABLE S4. Unadjusted and adjusted time-dependent (cancer diagnosis to COVID-19 diagnosis) Cox-regression analysis and all-cause mortality during Covid-period (January 15, 2020 and December 31, 2020)–Indiana, 2019-2020 (*N*=20,995)** | | | | | | |
| --- | --- | --- | --- | --- | --- | --- |
|  | **Unadjusted** | | | **Adjusted** | | |
| **Variable** | **Estimate (SE)** | **Hazard ratio**  **(95% CI)** | ***P*** | **Estimate (SE)** | **Hazard ratio**  **(95% CI)** | ***P*** |
| **Covid-diagnosis*** |  |  |  |  |  |  |
| No | -- | **Ref** | <.001 | -- | **Ref** | <.001 |
| Yes | 2.06 (0.09) | 7.88 (6.53-9.50) |  | 2.10 (0.09) | 8.20 (6.78-9.90) |  |
| **Age group, y** |  |  |  |  |  |  |
| 18—44 | -- | **Ref** |  | -- | **Ref** | <.001 |
| 45—64 | 1.00 (0.19) | 2.72 (1.87-3.96) |  | 1.00 (0.19) | 2.72 (1.87-3.96) |  |
| ≥65 | 1.50 (0.18) | 4.49 (3.12-6.45) |  | 1.44 (0.18) | 4.21 (2.91-6.10) |  |
| **Sex** |  |  |  |  |  |  |
| Female | -- | **Ref** | .01 | -- | **Ref** | <.01 |
| Male | 0.14 (0.05) | 1.15 (1.03-1.28) |  | 0.18 (0.06) | 1.20 (1.07-1.34) |  |
| **Race** |  |  |  |  |  |  |
| White | -- | **Ref** | 0.03 | -- | **Ref** | <.001 |
| Black | 0.06 (0.11) | 1.07 (0.86-1.32) |  | -0.01 (0.10) | 0.98 (0.79-1.22) |  |
| Hispanic | -0.49 (0.24) | 0.61 (0.38-0.99) |  | -0.41 (0.24) | 0.66 (0.41-1.07) |  |
| Other | -0.72 (0.10) | 0.49 (0.40-0.59) |  | -0.52 (0.10) | 0.59 (0.48-0.72) |  |
| **Comorbidities** |  |  |  |  |  |  |
| 0 | -- | **Ref** | <.001 |  | **Ref** | <.001 |
| 1 | 0.70 (0.07) | 2.01 (1.76-2.29) |  | 0.34 (0.07) | 1.40 (1.23-1.61) |  |
| ≥2 | 1.23 (0.07) | 3.42 (2.99-3.92) |  | 0.75 (0.07) | 2.12 (1.84-2.44) |  |
| **Cancer** |  |  |  |  |  |  |
| Other | -- | **Ref** | <.001 | -- | **Ref** | <.001 |
| Breast | -2.06 (0.18) | 0.12 (0.09-0.18) |  | -1.96 (0.18) | 0.14 (0.09-0.20) |  |
| Colorectal | -0.48 (0.13) | 0.61 (0.48-0.78) |  | -0.62 (0.12) | 0.54 (0.42-0.69) |  |
| Leukemia | -0.04 (0.13) | 0.96 (0.74-1.26) |  | -0.17 (0.13) | 0.84 (0.64-1.10) |  |
| Lip, oral cavity & pharynx | -0.56 (0.20) | 0.57 (0.38-0.85) |  | -0.63 (0.20) | 0.53 (0.35-0.79) |  |
| Lung and bronchus | 0.65 (0.08) | 1.91 (1.64-2.23) |  | 0.40 (0.08) | 1.49 (1.28-1.75) |  |
| Lymphoma | -0.47 (0.16) | 0.62 (0.46-0.84) |  | -0.49 (0.15) | 0.61 (0.46-0.83) |  |
| Myeloma | -0.22 (0.20) | 0.80 (0.53-1.19) |  | -0.52 (0.20) | 0.59 (0.40-0.88) |  |
| Other digestive | 0.67 (0.09) | 1.96 (1.65-2.32) |  | 0.45 (0.09) | 1.56 (1.31-1.86) |  |
| Other hematological | -1.44 (0.71) | 0.24 (0.06-0.95) |  | -1.64 (0.71) | 0.19 (0.05-0.78) |  |
| Prostate | -1.73 (0.16) | 0.18 (0.13-0.24) |  | -1.98 (0.16) | 0.14 (0.10-0.19) |  |
| Melanoma | -1.68 (0.16) | 0.18 (0.13-0.25) |  | -1.82 (0.16) | 0.16 (0.12-0.22) |  |
| Urinary tract | -0.53 (0.13) | 0.59 (0.46-0.76) |  | -0.79 (0.13) | 0.45 (0.35-0.58) |  |
| **Region** |  |  |  |  |  |  |
| Urban | -- | **Ref** | .63 | -- | **Ref** | .85 |
| Rural | -0.03 (0.06) | 1.03 (0.91-1.16) |  | -0.01 (0.06) | 1.01 (0.90-1.14) |  |

| **TABLE S5. Cancer subtype representation in the cancer cohort population, Indiana, 2019-2020.** | |
| --- | --- |
| **Cancer subtype** | **ICD-10 code** |
| Breast | C50 |
| Colorectal | C18-C21 |
| Leukemia | C91—C95 |
| Lip, oral cavity & pharynx | C00-C14 |
| Lung and bronchus | C34 |
| Lymphoma | C81-C85 |
| Myeloma | C90 |
| Other | C69-C72, C45-C49, C30-C39, C40-C41, C60-C63, C51-C58, C73-C75 |
| Other digestive^a^ (non-colorectal) | C15-C17, C22-C26 |
| Prostate | C61 |
| Melanoma | C43 |
| Urinary tract | C64-C68 |
| ^a^ Esophagus, stomach, small intestine, liver and intrahepatic bile ducts, gall bladder, and pancreas | |
